# 3D Bioprinting‐Assisted Engineering of Stem Cell‐Laden Hybrid Biopatches With Distinct Geometric Patterns Considering the Mechanical Characteristics of Regular and Irregular Connective Tissues

**DOI:** 10.1002/adhm.202502763

**Published:** 2025-07-07

**Authors:** Minjun Ahn, Gyu Tae Park, Arvind Kumar Shukla, Boguen Kwon, Jae Ho Kim, Eui‐Suk Sung, Byoung Soo Kim

**Affiliations:** ^1^ Medical Research Institute Pusan National University Yangsan 50612 Republic of Korea; ^2^ School of Biomedical Convergence Engineering Pusan National University Yangsan 50612 Republic of Korea; ^3^ Department of Physiology Pusan National University School of Medicine Yangsan 50612 Republic of Korea; ^4^ Department of Otolaryngology–Head and Neck Surgery, School of Medicine Pusan National University Yangsan 50612 Republic of Korea; ^5^ Research Institute for Convergence of Biomedical Science and Technology Pusan National University Yangsan Hospital Yangsan 50612 Republic of Korea

**Keywords:** 3D printing, geometric pattern, hybrid biopatch, mechanical behavior, soft connective tissue

## Abstract

Connective tissues display distinct mechanical behaviors, ranging from unidirectional stiffness in regular tissues to multidirectional compliance in irregular tissues. Replicating these biomechanical characteristics in engineered constructs remains a key challenge in regenerative medicine. This study presents a novel biofabrication platform for hybrid biopatches composed of tonsil‐derived mesenchymal stem cell (TMSC)‐laden collagen bioink reinforced with 3D printed polymeric patterns. Two distinct geometries, chiral and chevron, are designed to emulate the mechanical behavior of irregular and regular connective tissues, respectively. Mechanical testing shows that the chiral pattern exhibits quasi‐isotropic behavior with balanced stiffness and extensibility, whereas the chevron pattern demonstrate anisotropic mechanical properties. These mechanical features are maintained within the hybrid biopatches, leading to enhanced tensile strength and fatigue resistance compared with constructs composed solely of TMSC‐laden collagen. In a porcine mucosal defect model, the chiral‐patterned hybrid biopatch promoted superior epithelial repair, evidenced by narrower wound margins, continuous epithelial layers, and elevated expression of epithelial markers. These results suggest that mechanical compatibility with host tissue influences regenerative outcomes. Collectively, this study highlights the potential of incorporating geometric polymer patterns as a strategy for engineering tissue‐specific mechanics and improving regenerative performance, offering a promising platform for soft connective tissue repair.

## Graphical abstract

A hybrid biopatch platform integrating 3D printed polymeric patterns with stem cell‐laden collagen bioinks is developed to mimic the mechanical properties of connective tissues. By tailoring geometric architectures, the constructs replicate anisotropic or isotropic mechanics, enhancing tissue‐specific regeneration. In vivo studies show improved wound healing when mechanical compatibility aligns with the host tissue.

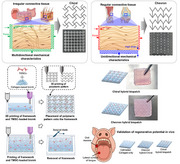

## 1 Introduction

Connective tissues in the human body exhibit diverse structural organizations and mechanical functions, depending on anatomical location and physiological role.^[^ ^1^ ^]^ Tissues such as tendons and ligaments, classified as regular connective tissues, contain highly aligned collagen fibers that enable unidirectional load‐bearing capacity.^[^ ^2^ ^]^ In contrast, irregular connective tissues, including the skin, oral mucosa, and dermis, consist of randomly oriented collagen networks that accommodate multidirectional mechanical stresses.^[^ ^3^ ^]^ These differences in fiber alignment play a critical role in determining tissue‐specific mechanical behaviors, including stiffness, elasticity, and tensile strength.^[^ ^1^, ^4^ ^]^ For instance, irregular connective tissues such as oral mucosa and dermis typically exhibit elastic moduli ranging from 10 to 100 kPa and tensile strengths of 5 to 50 kPa, depending on anatomical location and testing conditions.^[^ ^5^ ^]^ More importantly, these tissues are characterized by quasi‐isotropic mechanical behavior due to their randomly oriented collagen networks. In contrast, regular connective tissues such as tendons display pronounced anisotropy, with elastic moduli of 500–1500 MPa along the fiber direction and significantly lower values (20–50 MPa) in the transverse direction, as well as ultimate tensile strengths of 50–150 MPa.^[^ ^5^, ^6^ ^]^ This stark directional dependence highlights the importance of mimicking mechanical anisotropy when designing tissue‐specific implants.

Mechanical compatibility between engineered constructs and host tissues has emerged as a key factor influencing regenerative success.^[^ ^7^ ^]^ A growing body of evidence shows that stem cell fate, extracellular matrix (ECM) remodeling, and wound healing are regulated not only by biochemical cues but also by mechanical properties such as substrate stiffness and dynamic strain.^[^ ^8^ ^]^ As a result, the design of engineered bioconstructs that replicate the mechanical properties of target tissues has gained increasing importance in regenerative medicine.

Incorporating stem cells into bioconstructs is also essential for effective tissue regeneration. Stem cells contribute to healing by differentiating into tissue‐specific lineages and by secreting bioactive molecules that modulate inflammation, stimulate angiogenesis, and induce endogenous repair mechanisms.^[^ ^7^, ^9^ ^]^ These paracrine effects are particularly beneficial in tissue engineering applications, where both structural integration and functional recovery are desired. However, the regenerative efficacy of stem cell‐laden constructs depends on maintaining stemness within the engineered microenvironment.^[^ ^10^ ^]^ Upon encapsulation in hydrogels or other biomaterials, stem cells may undergo phenotypic drift or premature differentiation. Thus, establishing a supportive environment that preserves stemness during both in vitro culture and post‐implantation is critical.

To enhance structural stability while maintaining biological functionality, hybrid biopatches combining bioactive hydrogels with mechanically reinforced synthetic polymer substrates have been proposed.^[^ ^11^ ^]^ Advances in 3D printing have enabled new fabrication strategies for such hybrid systems.^[^ ^7^, ^9^, ^12^ ^]^ Although synthetic polymers provide superior printability and mechanical strength for building complex architectures, their application in soft connective tissue regeneration remains limited. Challenges persist in replicating the compliant and anisotropic mechanics of native tissues and achieving stable integration between polymeric frameworks and cell‐laden bioinks. Furthermore, the influence of tissue‐specific mechanical mimicry on regenerative outcomes, particularly in soft connective tissues, remains poorly understood.

In this study, we emphasize not only the mechanical magnitude but also the directional dependence of mechanical properties, by mimicking the anisotropic and isotropic architectures of regular and irregular connective tissues, respectively, through geometry‐guided polymeric patterning. A hybrid biopatch was developed by integrating tonsil‐derived mesenchymal stem cell (TMSC)‐laden collagen bioinks with 3D printed polymeric patterns of distinct geometries. By evaluating the mechanical performance of chiral and chevron architectures, designed to mimic the properties of irregular and regular connective tissues, respectively, this study aimed to determine whether mechanical compatibility between engineered constructs and host tissues enhances regeneration. Furthermore, a porcine full‐thickness mucosal defect model was used to assess the in vivo regenerative efficacy of these patterned biopatches. This work proposes a new design paradigm for developing mechanically responsive, tissue‐specific implants for soft connective tissue repair.

## 2 Results

### 2.1 3D Printing of Mechanically Tunable Polymeric Patterns

**Figure 1:**
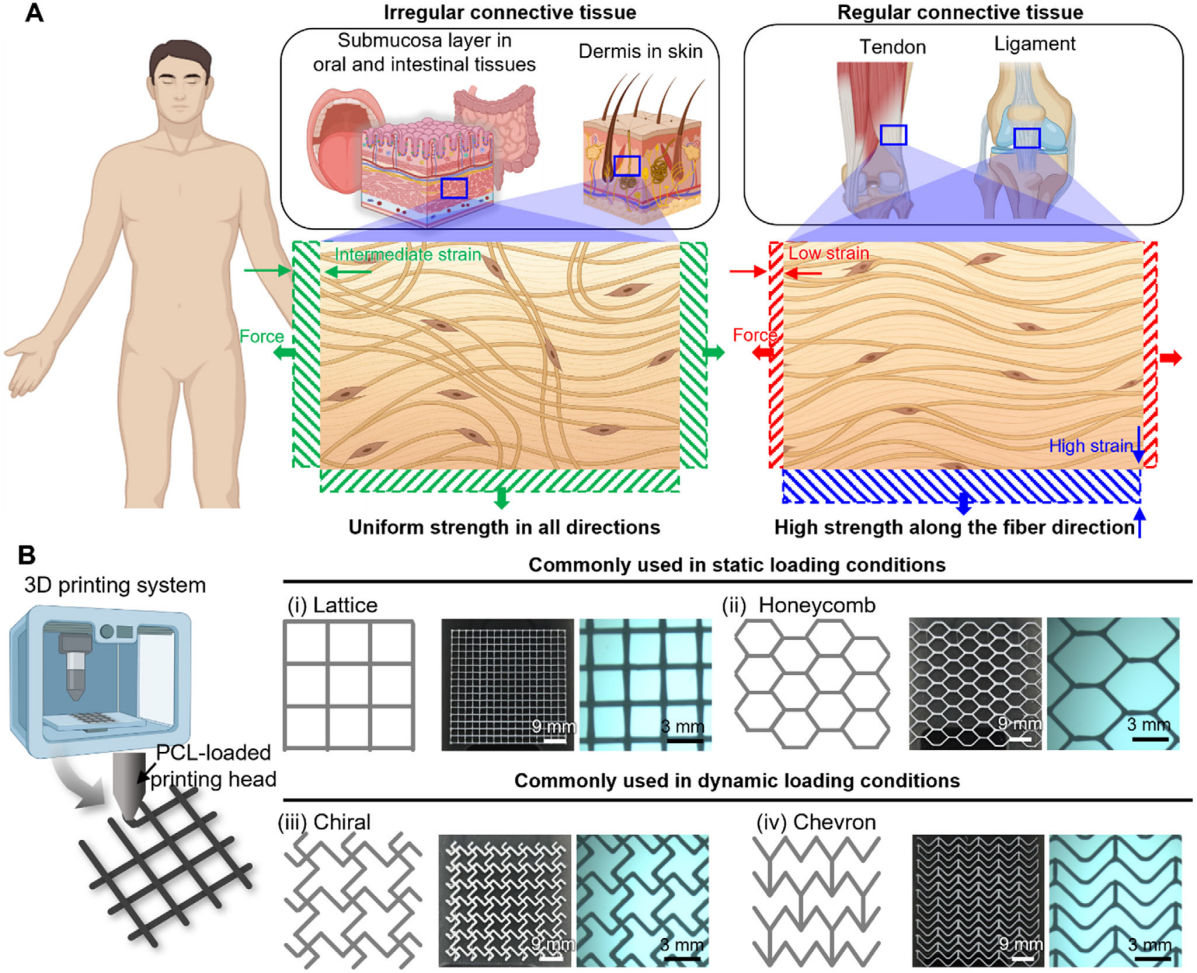
Overview of connective tissue mechanics and 3D printing of mechanically tunable polymeric patterns. A) Schematic illustration showing examples of irregular and regular connective tissues in the human body. Tissue‐specific mechanical characteristics are determined by the orientation of fiber alignment. B) Representative 3D printed geometric patterns commonly applied under static or dynamic mechanical conditions. Polycaprolactone (PCL) was used to fabricate four distinct patterns: (i) lattice, (ii) honeycomb, (iii) chiral, and (iv) chevron. Each pattern exhibits unique architectural features and degrees of structural anisotropy.

Connective tissues in the human body display diverse mechanical behaviors primarily dictated by the orientation and organization of collagen fibers (**Figure** **1**A). To replicate these tissue‐specific mechanical characteristics, four distinct geometric patterns were designed and fabricated via extrusion‐based 3D printing using the biodegradable synthetic polymer polycaprolactone (PCL) (Figure 1B). Lattice and honeycomb structures were selected as representative geometries commonly used under static loading conditions. The lattice pattern consisted of repeating orthogonal linear elements forming a grid‐like architecture (Figure 1B(i)), whereas the honeycomb pattern comprised contiguous hexagonal units, structurally inspired by natural honeycombs (Figure 1B(ii)). For dynamic loading applications, chiral and chevron patterns were employed due to their unique mechanical properties. The chiral pattern featured repeating symmetric units arranged without global mirror symmetry (Figure 1B(iii)). This configuration allows for auxetic behavior and consistent mechanical responses in both horizontal and vertical directions. In contrast, the chevron pattern consisted of repeating V‐shaped elements aligned in a uniform direction, promoting directional stiffness and tensile strength along the primary alignment axis, while exhibiting contrasting mechanical behavior in the orthogonal direction (Figure 1B(iv)). To ensure fair mechanical comparison, all patterns were fabricated using identical PCL mass, thereby standardizing the material input across groups (Figure S1, Supporting Information).

### 2.2 Mechanical Analysis of 3D Printed Polymeric Patterns

**Figure 2:**
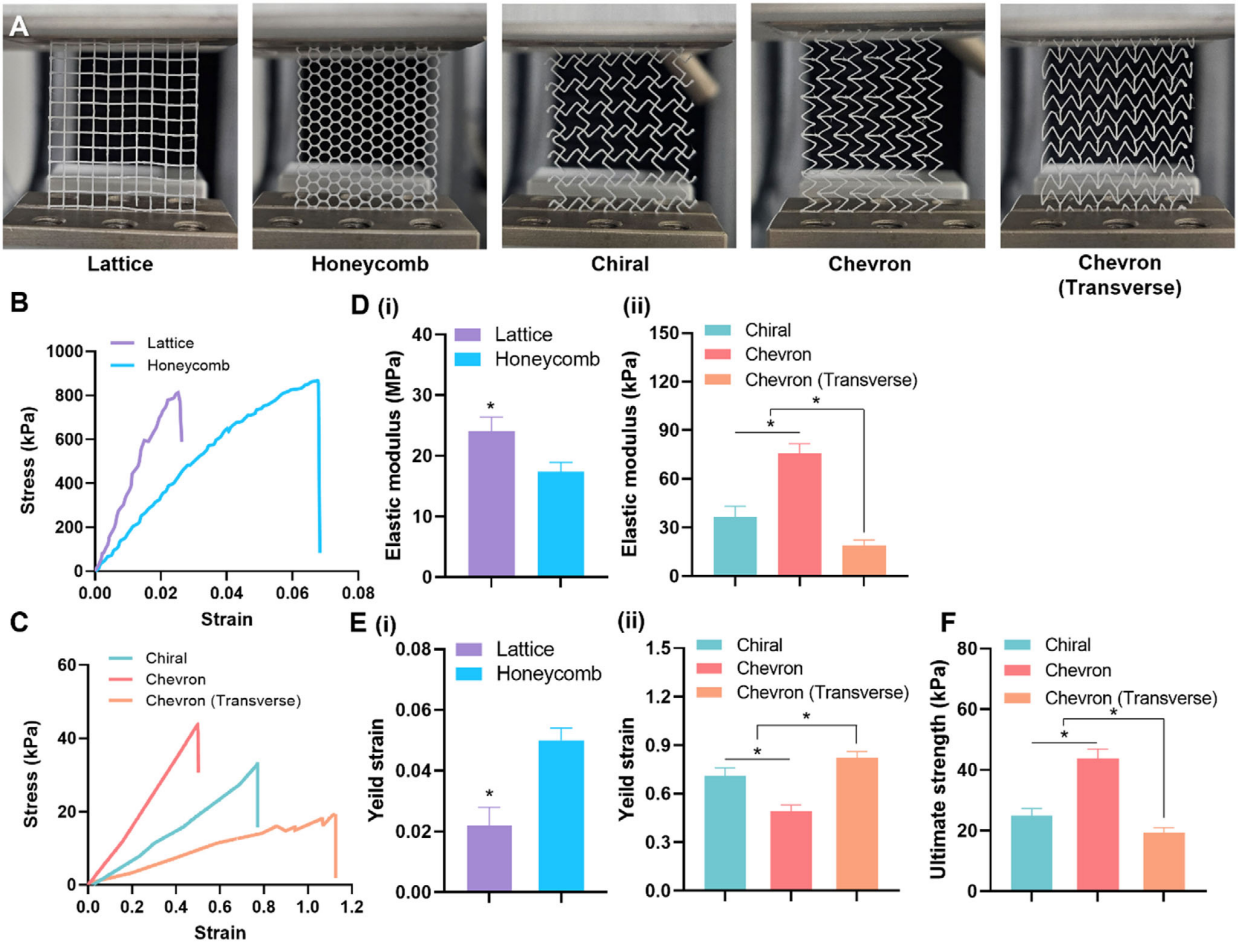
Tensile mechanical characterization of 3D printed polymeric patterns. A) Photographs of 3D printed polymeric patterns mounted on a universal testing machine for mechanical testing. Stress–strain curves obtained from tensile testing of B) static patterns (lattice and honeycomb) and C) dynamic patterns (chiral, chevron, and chevron transverse). D) Quantification of elastic modulus for (i) static and (ii) dynamic groups (*p < 0.05). E) Yield strain values of the (i) static and (ii) dynamic groups (*p < 0.05). F) Ultimate tensile strength of each pattern (*p < 0.05). All values are presented as mean ± standard deviation (SD).

To evaluate the mechanical performance of the 3D printed patterns, uniaxial tensile and cyclic loading tests were conducted on five geometries: lattice, honeycomb, chiral, chevron, and a 90°‐rotated chevron (referred to as chevron (transverse)) (**Figure** **2**A). Due to its symmetric design, the chiral pattern was assumed to exhibit similar mechanical behavior under loading in both the horizontal and vertical directions.

Tensile stress–strain analysis revealed substantial differences in mechanical response depending on pattern architecture. The lattice and honeycomb patterns, commonly associated with static mechanical environments, exhibited steep stress–strain curves with limited deformability (Figure 2B). Fracture occurred at strains of 0.0262 ± 0.0054 for the lattice and 0.0656 ± 0.0038 for the honeycomb pattern, reflecting their high stiffness and brittle mechanical nature. In contrast, the chiral, chevron, and chevron (transverse) patterns exhibited higher fracture strain values, 0.4885 ± 0.0254, 0.7658 ± 0.0318, and 1.2428 ± 0.0388, respectively, indicating superior elasticity and compliance (Figure 2C).

Quantitative analysis of the tensile curves further confirmed these trends (Figure 2D–,F). As expected, the lattice and honeycomb patterns showed the highest elastic modulus values (24.04 ± 2.35 MPa and 17.38 ± 1.52 MPa, respectively), consistent with their stiff, load‐resisting architectures (Figure 2D(i)). Among the patterns designed for dynamic conditions, the chevron pattern exhibited the highest elastic modulus (75.88 ± 5.80 kPa), followed by chiral (36.76 ± 6.4 kPa) and chevron (transverse) ones (19.08 ± 3.2 kPa) (Figure 2D(ii)). Yield strain measurements followed a similar trend (Figure 2E). The lattice and honeycomb structures showed low yield strain values (0.022 ± 0.006 and 0.050 ± 0.004, respectively) (Figure 2E(i)), indicating limited extensibility prior to plastic deformation. In contrast, the chevron (transverse) pattern showed the highest yield strain (0.820 ± 0.040), followed by chiral (0.709 ± 0.050) and chevron (0.492 ± 0.041) (Figure 2E(ii)). These results emphasize the direction‐dependent mechanical properties of the chevron architecture: rotation by 90° results in enhanced stretchability and reduced stiffness. The ultimate tensile strength also varied significantly between dynamic patterns (Figure 2F). The chevron pattern exhibited the highest strength (43.8 ± 3.1 kPa), while the chiral (25.0 ± 2.3 kPa) and chevron (transverse) (19.2 ± 1.8 kPa) patterns demonstrated lower values. These findings indicate that alignment of the chevron pattern along the loading direction increases load‐bearing capacity, whereas the rotated configuration enhances flexibility.

**Figure 3:**
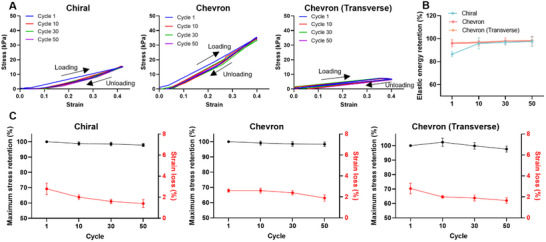
Cyclic evaluation of 3D printed polymeric patterns. A) Cyclic stress–strain curves over 50 loading‐unloading cycles. B) Elastic energy retention over 50 cycles for each group. sC) Maximum stress retention and cumulative strain loss throughout cyclic loading. All values are presented as mean ± standard deviation (SD).

To assess mechanical durability, cyclic tensile tests were conducted on the chiral, chevron, and chevron (transverse) patterns over 50 loading‐unloading cycles within the elastic range (strain < 0.4). All three designs maintained stable stress–strain profiles throughout the cycles, indicating strong mechanical resilience (**Figure** **3**A). Elastic energy retention was consistently high across the groups, with both chevron and chevron (transverse) patterns retaining over 95% of input energy (Figure 3B). Although the chevron (transverse) group exhibited slightly greater hysteresis during the first cycle, performance stabilized in subsequent cycles. Further analysis of maximum stress and cumulative strain loss confirmed minimal mechanical degradation in all patterns (Figure 3C). Maximum stress retention remained above 97%, and cumulative strain loss after 50 cycles was below 3%, with no statistically significant differences among the groups. These results confirm the mechanical stability of the dynamically tuned designs under prolonged cyclic loading, supporting their applicability in dynamic tissue environments.

Collectively, these findings demonstrate that geometric patterning provides a powerful strategy for modulating mechanical behavior in printed biomaterials. In particular, the chiral and chevron patterns exhibit unique yet complementary mechanical profiles that correspond to the behaviors of irregular and regular connective tissues, respectively, thereby establishing their utility as structural analogs for tissue‐specific applications.

### 2.3 Establishment of Environmental Bioink Conditions for Tonsil‐Derived Stem Cells

**Figure 4:**
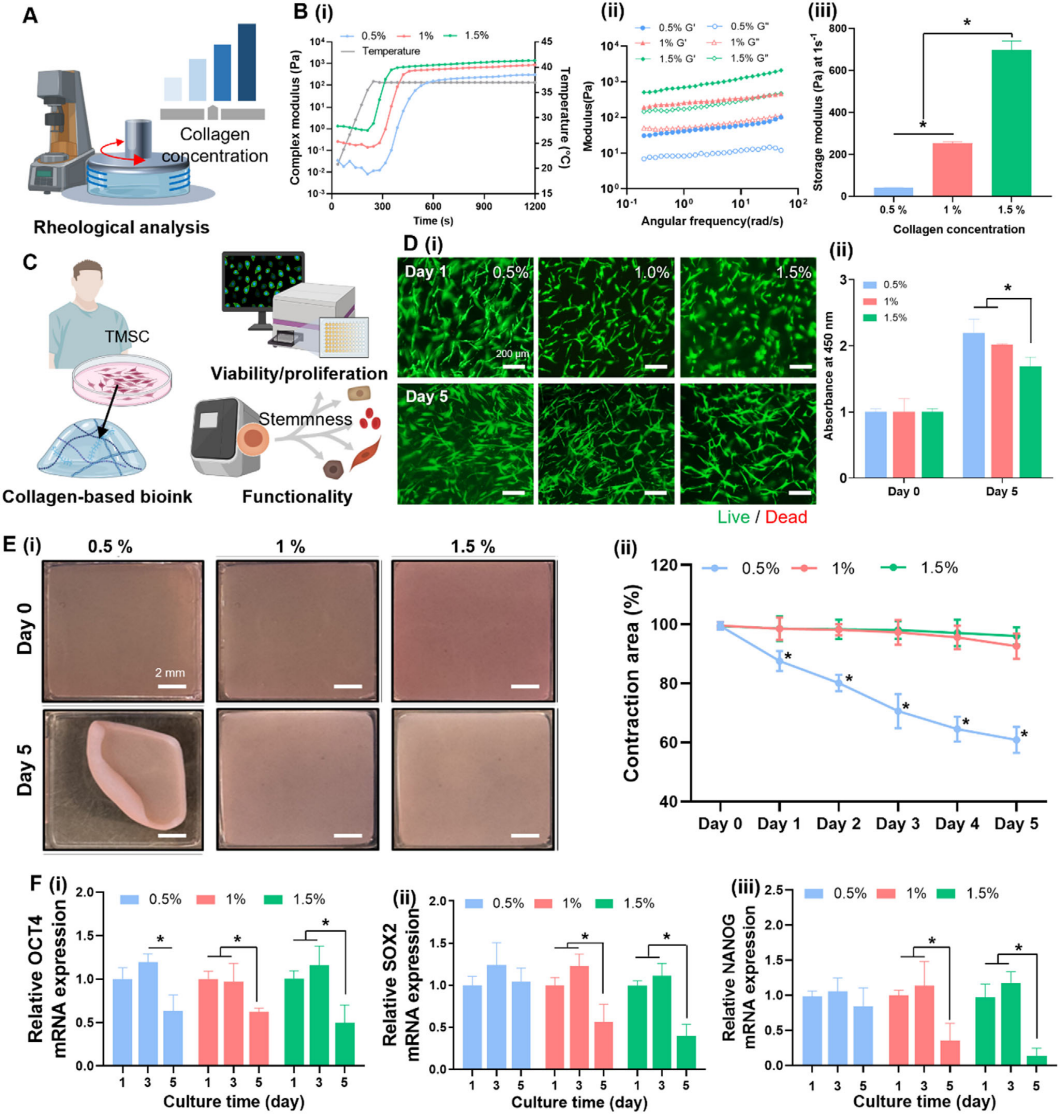
Establishment of bioink and culture conditions for tonsil‐derived mesenchymal stem cells (TMSCs). A) Schematic illustration of rheological analysis performed on collagen‐based bioinks with varying concentrations (0.5%, 1.0%, and 1.5%). B) Rheological properties of the collagen bioinks: (i) Gelation kinetics measured from 15 °C to 37 °C, (ii) frequency sweep showing storage (G′) and loss (G′′) moduli over a range of 0.1–100 rad s^−1^ after gelation, and (iii) storage modulus at 1 rad s^−1^ (*p < 0.05). C) Schematic overview of procedures for evaluating TMSC viability, proliferation, and functionality in collagen‐based bioinks. D) (i) Live/dead staining of TMSCs on days 1 and 5. Scale bars: 200 µm. (ii) Cell proliferation assessed using a CCK‐8 assay. (*p < 0.05). E) Contraction behavior of TMSC‐laden bioinks: (i) Representative photographs of constructs composed of 0.5%, 1.0%, and 1.5% collagen bioinks at day 0 and day 5. Scale bars: 2 mm. (ii) Quantification of the retained construction area. *p < 0.05 compared to the 0.5% and 1.0% groups. (F) qRT‐PCR analysis of stemness‐related gene expression levels: (i) *OCT4*, (ii) *SOX2*, and (iii) *NANOG*. *p < 0.05.

TMSCs, known for their high efficacy in mucosal regeneration, were selected as the cell source for in vivo regenerative assessment in this study.^[^ ^13^ ^]^ To optimize the biophysical environment for TMSCs, collagen‐based bioinks of varying concentrations (0.5%, 1.0%, and 1.5%) were prepared and evaluated for their rheological and biological properties (**Figure** **4**A).

Rheological analysis confirmed that all tested bioinks underwent successful temperature‐induced gelation at 37 °C (Figure 4B(i)), indicating suitability for physiological crosslinking. Gelation kinetics revealed that higher collagen concentrations resulted in faster gelation: 1.5% bioink gelled within 387 ± 5.4 s, 1.0% within 493 ± 3.8 s, and 0.5% within 669 ± 3.1 s. Frequency sweep measurements post‐gelation showed that both storage (G′) and loss (G″) moduli increased with collagen concentration, with the 1.5% formulation exhibiting the highest viscoelastic properties (Figure 4B(ii)). In all groups, G′ values consistently exceeded G″ values across the full range of angular frequencies, indicating elastic‐dominant behavior. Quantification at 1 rad s^−1^ showed significant stiffness enhancement with increasing concentration: storage moduli values of 41.3 ± 0.23 Pa for 0.5%, 252.2 ± 8.9 Pa for 1.0%, and 697.8 ± 43.1 Pa for 1.5% bioinks (Figure 4B(iii)).

To assess biocompatibility, TMSCs were encapsulated in each bioink and analyzed for viability, proliferation, and functional behavior (Figure 4C). Live/dead staining indicated uniformly high viability (> 95%) across all concentrations on both day 1 and 5 (Figure 4D(i)). Cell proliferation also remained consistent across groups, with no statistically significant differences observed on day 5 (Figure 4D(ii)), confirming that all tested bioinks support short‐term TMSC viability and growth. Next, the structural integrity of TMSC‐laden constructs was assessed by measuring gel contraction over 5 days (Figure 4E(i)). Although 1.0% and 1.5% bioinks retained their original dimensions, the 0.5% group exhibited significant shrinkage. Retention of the contraction area was 60.8% **± 4.4%** for 0.5% bioink, significantly lower than those of 1.0% (92.5% ± 4.3%) and 1.5% (96.0% ± 2.9%) formulations (Figure 4E(ii)). These results indicate that although all concentrations supported viability and proliferation, a minimum of 1.0% collagen was required to prevent excessive gel contraction and preserve construct integrity during short‐term culture.

Lastly, stemness‐associated gene expression was evaluated using quantitative reverse transcription‐PCR (qRT‐PCR) by using the canonical stemness markers (*OCT4*, *SOX2*, and *NANOG*) (Figure 4F). Expression levels were largely maintained on day 3, with no significant differences from those of day 1. However, all groups showed a notable decline in expression by day 5, with levels falling below 40% relative to those of day 1. These results suggest that TMSC identity is retained for up to 3 days in collagen‐based bioinks, after which stemness significantly diminishes. Based on these results, a collagen concentration of 1.0% and a 3‐day culture window were selected for all subsequent experiments to ensure optimal structural and biological performance.

### 2.4 Biofabrication of Polymeric Pattern‐Integrated Hybrid Biopatches

**Figure 5:**
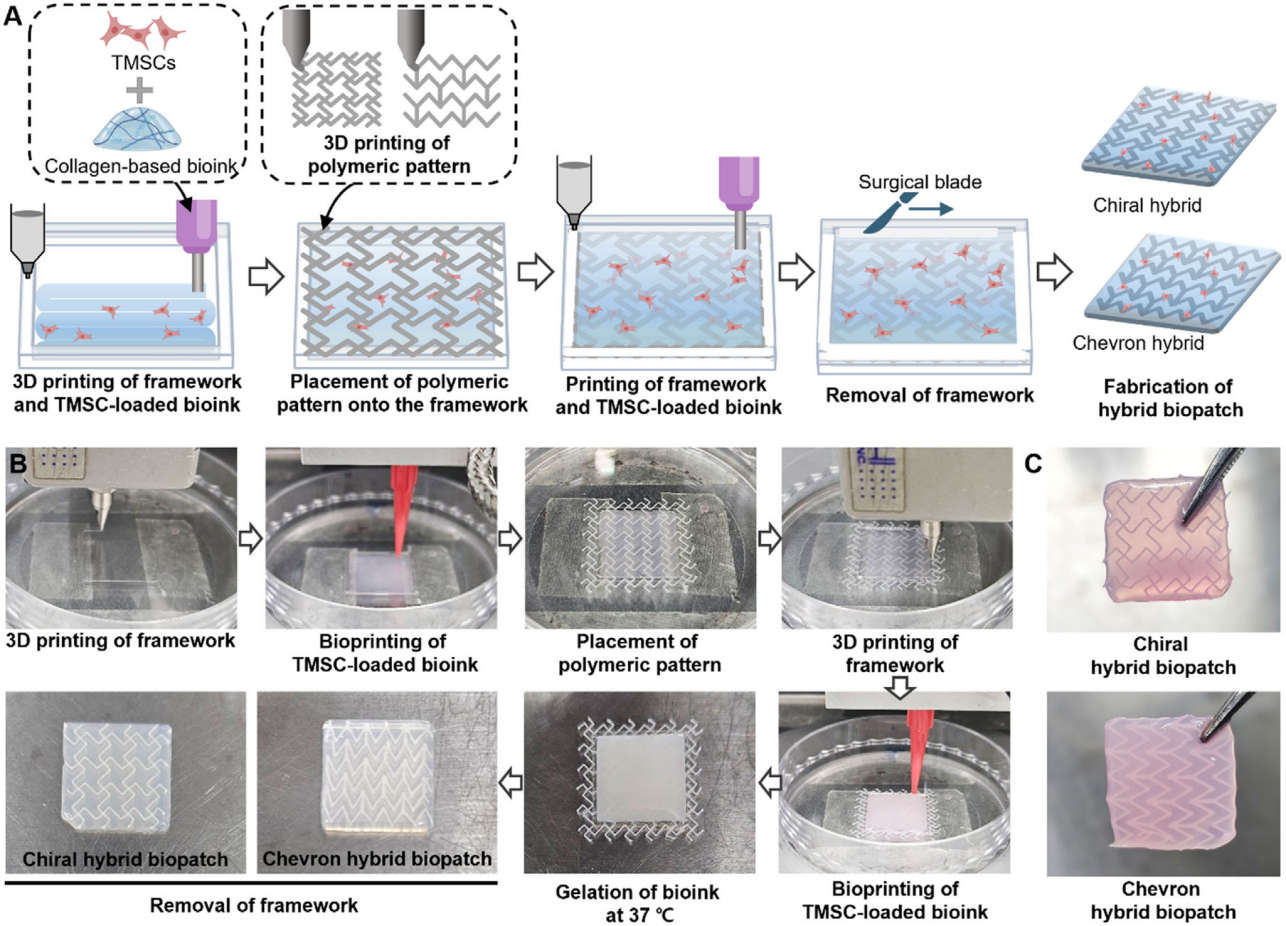
Biofabrication of TMSC‐laden hybrid biopatches incorporating geometric polymeric patterns. A) Schematic illustration of the fabrication process for hybrid biopatches. A PEVA‐based framework is first printed onto a Petri dish, followed by the deposition of a TMSC‐laden collagen bioink within the framework. A prefabricated polymeric pattern (chiral or chevron) is then positioned onto the construct. Additional PEVA walls and bioink layers are sequentially printed to fully embed the pattern. After thermal gelation at 37 °C for 30 min, the PEVA mold is removed using a surgical blade, yielding a freestanding hybrid biopatch. B) Sequential photographs depicting the biofabrication process from PEVA mold printing to the final creation of chiral and chevron hybrid biopatches. C) Photographs showing the robust handling ability of the resulting hybrid biopatches using forceps, confirming structural integrity and cohesion.

To engineer tissue‐specific constructs with biomimetic mechanical performance and sustained stem cell functionality, a hybrid biopatch was developed by integrating geometrically predefined polymeric patterns with TMSC‐laden collagen bioink (**Figure** **5**A). Based on previous mechanical analyses, chiral and chevron patterns were selected to emulate the properties of irregular and regular connective tissues, respectively. Concurrently, the optimized 1.0% collagen bioink formulation was used to encapsulate TMSCs, ensuring both cellular viability and matrix stability.

The fabrication process began with 3D printing of a polyethylene vinyl acetate (PEVA) framework onto a Petri dish, forming a mold to contain the bioink (Figure 5B). The TMSC‐laden collagen bioink was then dispensed within the PEVA boundary. A prefabricated polymeric pattern, either chiral or chevron, was carefully positioned atop of the bioink‐filled structure. To fully encapsulate the pattern within the hybrid biopatch, an additional PEVA wall and a second layer of bioink were sequentially printed. The construct was then incubated at 37 °C for 30 min to induce thermal gelation of the collagen matrix. After gelation, the PEVA framework was mechanically removed using a surgical blade, yielding a freestanding hybrid biopatch embedded with a mechanically distinct polymeric pattern.

The resulting hybrid biopatches retained their fabricated shape without significant contraction over 3 days (Figure S2, Supporting Information). They also maintained structural integrity and exhibited excellent handling properties, as confirmed through forceps manipulation without damage or delamination (Figure 5C). Both chiral and chevron‐type biopatches were successfully produced using this modular assembly approach. This fabrication strategy enables the precise spatial integration of pre‐characterized mechanical architectures with viable cell‐laden bioinks, establishing a robust and adaptable platform for engineering biologically active, tissue‐mimetic soft tissue patches.

### 2.5 Mechanical Characterization of Hybrid Biopatches Incorporating Polymeric Patterns

**Figure 6:**
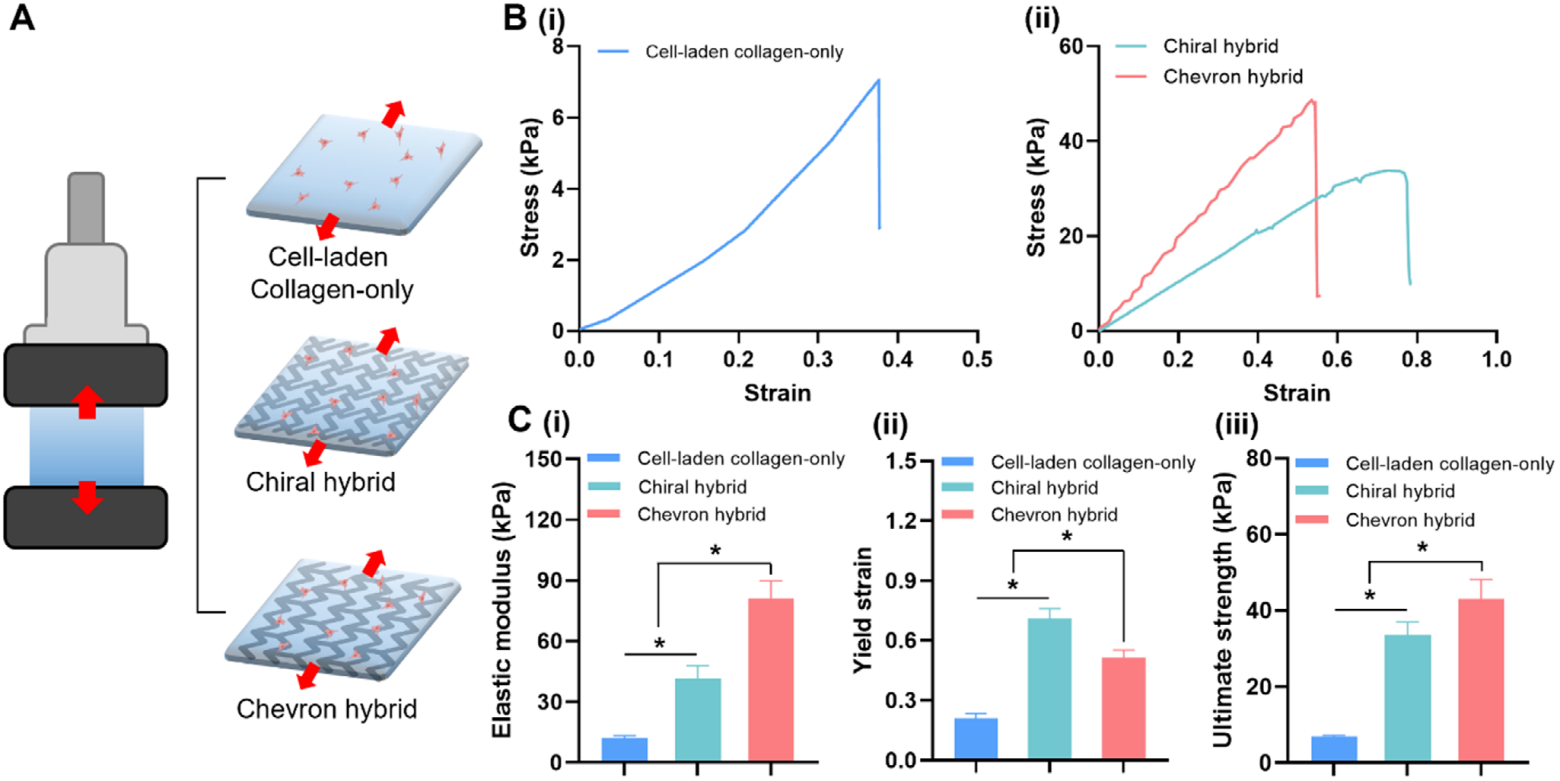
Tensile mechanical evaluation of hybrid biopatches incorporating polymeric patterns. A) Schematic of the uniaxial tensile testing setup for three groups: a cell‐laden collagen‐only biopatch (no polymer), and hybrid biopatches containing either a chiral or chevron pattern. B) Representative tensile stress–strain curves for the (i) collagen‐only and (ii) hybrid biopatches. C) Quantitative comparison of (i) elastic modulus, (ii) yield strain, and (iii) ultimate tensile strength. *p < 0.05. Values are presented as mean ± standard deviation (SD).

To evaluate the mechanical reinforcement provided by embedded polymeric patterns, three experimental groups were compared: 1) a biopatch composed solely of TMSC‐laden collagen bioink (cell‐laden collagen‐only), 2) a chiral pattern hybrid biopatch (chiral hybrid), and 3) a chevron pattern hybrid biopatch (chevron hybrid) (**Figure** **6**A). Uniaxial tensile and cyclic loading tests were conducted to assess mechanical performance.

In tensile testing, the cell‐laden collagen‐only biopatch displayed a low‐gradient stress–strain curve, typical of soft hydrogels, indicating low stiffness and limited load‐bearing capacity (Figure 6B(i)). In contrast, both hybrid biopatches exhibited steeper stress–strain profiles (Figure 6B(ii)), reflecting the mechanical reinforcement provided by the polymeric patterns. These curves confirm that the intrinsic mechanical features of each pattern were retained after integration into the hydrogel matrix. Quantitative analysis supported these findings. The cell‐laden collagen‐only biopatch exhibited the lowest mechanical metrics: elastic modulus (12.1 ± 1.3 kPa), yield strain (0.21 ± 0.02), and tensile strength (7.05 ± 0.24 kPa) (Figure 6C). The chevron hybrid biopatch exhibited the highest elastic modulus (81.2 ± 8.5 kPa) and tensile strength (43.1 ± 5.1 kPa), whereas the chiral hybrid demonstrated a more balanced profile, characterized by higher extensibility (yield strain: 0.71 ± 0.05), moderate stiffness (modulus: 41.4 ± 6.5 kPa), and intermediate tensile strength (33.7 ± 3.3 kPa). These results reaffirm that the distinct mechanical behaviors of the chiral and chevron patterns were preserved in the hybrid constructs.

**Figure 7:**
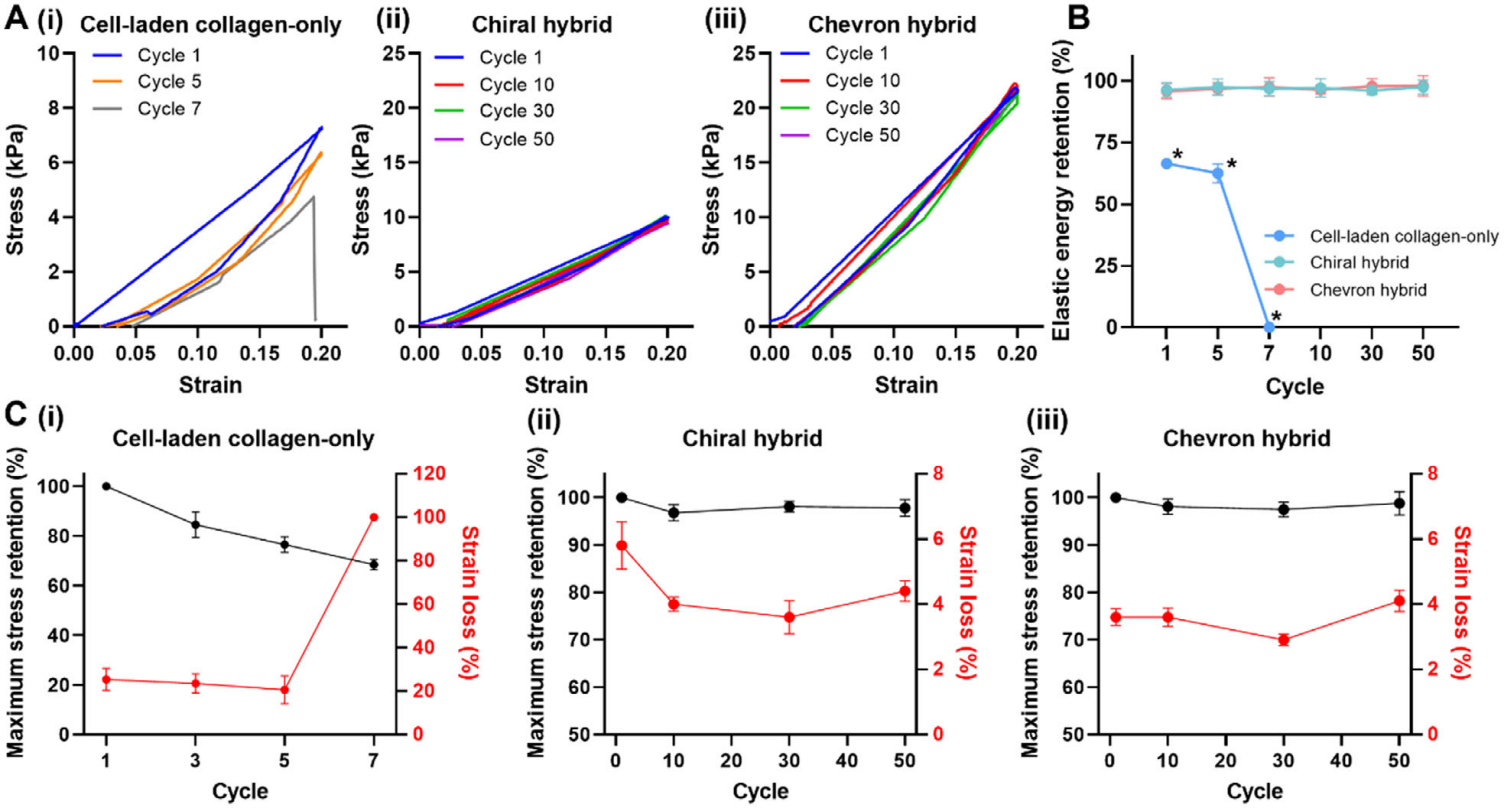
Cyclic evaluation of hybrid biopatches incorporating polymeric patterns. A) Cyclic stress–strain curves over 50 loading‐unloading cycles for the (i) collagen‐only, (ii) chiral hybrid, and (iii) chevron hybrid biopatches. B) Elastic energy retention across 50 cycles. *p < 0.05 compared to the hybrid groups. C) Analysis of maximum stress retention and cumulative strain loss during cyclic loading for the (i) collagen‐only, (ii) chiral hybrid, and (iii) chevron hybrid biopatches. Values are presented as mean ± standard deviation (SD).

Cyclic testing was performed to evaluate fatigue resistance (**Figure** **7**A). The cell‐laden collagen‐only biopatch showed progressive mechanical degradation, with increased hysteresis and decreased stress response over successive cycles. Fracture occurred by cycle 7, indicating poor durability (Figure 7A(i)). In contrast, both hybrid biopatches maintained consistent stress–strain behavior over 50 cycles, demonstrating high mechanical resilience (Figure 7A(ii, iii)). Elastic energy retention exceeded 96% throughout all cycles for both hybrid groups, whereas the cell‐laden collagen‐only biopatch exhibited a sharp decline in energy retention and cumulative strain loss prior to fracture (Figure 7B).

Further analysis revealed that in the cell‐laden collagen‐only biopatch, maximum stress retention declined progressively, falling to 68.4% by the seventh cycle, at which point structural failure occurred (Figure 7C(i)). Additionally, strain loss exceeded 20% per cycle, indicating poor elastic recovery and extensive plastic deformation under repeated loading. Conversely, both the chiral and chevron hybrid biopatches maintained excellent mechanical stability over 50 cycles (Figure 7C(ii, iii)). Maximum stress retention remained high, 96% for the chiral hybrid and 97% for the chevron hybrid, without significant deviation from those of the initial cycle. Strain loss in both groups consistently remained below 5%, confirming robust mechanical resilience and effective elastic recovery. These results demonstrate that the incorporation of reinforcing polymeric patterns significantly enhances the fatigue resistance of soft hydrogel constructs subjected to cyclic mechanical stress.

Taken together, these findings indicate that the integration of mechanically pre‐characterized polymeric patterns into stem cell‐laden bioinks significantly improves both the load‐bearing capacity and fatigue resistance of hydrogel constructs. Furthermore, the distinct mechanical responses observed between the chiral and chevron hybrid biopatches validate that the pattern‐specific mechanical behaviors established during earlier evaluations were faithfully preserved within the hybrid architecture.

### 2.6 Validation of Regenerative Potential of Mechanically Tunable Hybrid Biopatches in Vivo

**Figure 8:**
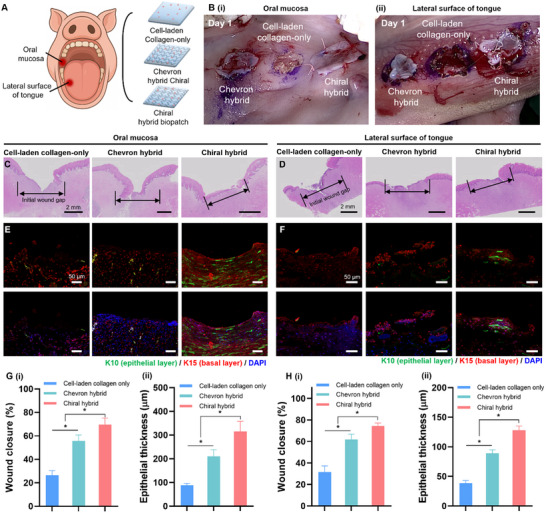
Evaluation of the regenerative potential of hybrid biopatches mimicking the mechanical characteristics of regular and irregular connective tissues. A) Schematic of the surgical implantation of three experimental groups into full‐thickness mucosal defects in the oral cavity and lateral tongue of a porcine model. B) Postoperative photographs on day 2 showing retention of the hybrid biopatches at the implantation sites, compared with detachment of the collagen‐only construct. C) H&E‐stained sections of harvested oral mucosa and D) lateral tongue tissues on day 14. Black arrows indicate the initial wound gaps. Scale bars: 2 mm. E) Immunofluorescence staining of oral mucosa sections and F) lateral tongue sections for cytokeratin 10 (K10, epithelial layer; green) and cytokeratin 15 (K15, basal layer; red), with DAPI nuclear counterstaining (blue). Scale bars: 50 µm. Quantitative analysis of G) oral mucosa and H) lateral tongue, including (i) wound closure rate and (ii) epithelial thickness. *p < 0.05. Values are presented as mean ± standard deviation (SD).

This study hypothesized that a biopatch mimicking the mechanical behavior of the host tissue could provide a more favorable environment for tissue repair. To test this hypothesis, full‐thickness mucosal defects were created in a porcine model at two anatomical sites: the inner cheek (oral mucosa) and the lateral surface of the tongue (**Figure** **8**A). Regenerative efficacy was assessed across three experimental groups: 1) cell‐laden collagen‐only biopatch, 2) chiral hybrid biopatch‐treated defects, and 3) chevron hybrid biopatch‐treated defects. Postoperative imaging confirmed that the cell‐laden collagen‐only biopatch detached from the implantation site by day 1, whereas both hybrid biopatches remained securely in place (Figure 8B). This detachment was likely due to poor mechanical coherence between the soft hydrogel and host tissue, even when sutured.

Histological analysis of explanted tissues on day 14 was conducted to assess epithelial regeneration and structural restoration. Hematoxylin and eosin (H&E) staining of oral mucosa samples showed narrower residual wound gaps in the chiral hybrid group than in the cell‐laden collagen‐only and chevron hybrid groups (Figure 8C). Similarly, tongue tissue from the chiral group exhibited greater epithelial continuity and reduced defect width compared to those in other groups (Figure 8D). Immunofluorescence staining for epithelial (K10) and basal layer (K15) markers provided further insight into re‐epithelialization. In both oral mucosa and tongue tissues, the chiral hybrid group exhibited more robust and continuous K10‐positive epithelial layers, as well as stronger K15‐positive basal layer formation, compared to the other two groups (Figure 8E, F). The chevron hybrid biopatch also supported tissue coverage, though to a lesser degree, whereas the collagen‐only group showed discontinuous epithelial margins and sparse basal layer restoration. Quantitative analysis of wound healing further supported these histological findings (Figure 8G, H). In the oral mucosa, the wound closure rate was highest in the chiral hybrid group (69.7 ± 5.5%), followed by the chevron hybrid group (55.8 ± 5.0%), and the collagen‐only group (26.5 ± 3.9%) (Figure 8G(i)). A similar trend was observed in epithelial thickness, with the chiral hybrid group exhibiting the thickest epithelium (315 ± 43 µm), followed by the chevron (210 ± 28 µm) and collagen‐only (88 ± 8 µm) groups (Figure 8G(ii)).

In the lateral tongue, the chiral hybrid group again demonstrated superior wound closure (74.3 ± 2.8%), compared to chevron (61.8 ± 5.0%) and collagen‐only (31.5 ± 5.8%) groups (Figure 8H(i)). The epithelial thickness also followed this pattern, with values of approximately 128.1 ± 7.1 µm in the chiral group, 89.2 ± 5.7 µm in the chevron group, and 38.5 ± 4.5 µm in the collagen‐only group (Figure 8H(ii)).

These findings provide preliminary evidence supporting the hypothesis that mechanical compatibility between a biopatch and its host environment may enhance structural repair, particularly in mechanically dynamic connective tissues.

## 3 Discussion

This study presents a novel platform for engineering stem cell‐laden hybrid biopatches by integrating 3D printed polymeric patterns designed to emulate the mechanical characteristics of regular and irregular connective tissues. By demonstrating clear mechanical distinctions between chiral and chevron patterns, our findings underscore the pivotal role of geometric patterning in engineering constructs with tissue‐specific mechanical functionality and regenerative potential.^[^ ^7^, ^14^ ^]^

The chiral pattern exhibited balanced mechanical performance, characterized by moderate elastic modulus, tensile strength, and yield strain compared with those of the chevron pattern. This quasi‐isotropic response closely resembles the behavior of irregular connective tissues, which must accommodate multiaxial stress during physiological activity. These results support the utility of chiral architectures as structural analogs for mechanically compliant, multidirectionally responsive soft tissues.^[^ ^15^ ^]^ In contrast, the chevron pattern effectively mimicked the anisotropic mechanics of regular connective tissues such as tendons and ligaments, exhibiting high stiffness and tensile strength along its primary axis. Upon 90° rotation, the chevron pattern demonstrated reduced stiffness and increased stretchability, confirming its directional mechanical dependence. This tailored anisotropy may be leveraged to guide cell alignment, ECM deposition, and functional tissue remodeling, consistent with the structurally adaptive nature of native connective tissues subjected to directional loading.^[^ ^1^, ^3^ ^]^

A critical advancement in this study was the successful integration of mechanically distinct polymeric frameworks within soft hydrogel matrices while preserving the intrinsic mechanical properties of each pattern. This approach overcomes key limitations of conventional strategies, such as casting or printing onto stiff polymer grids, which often yield mechanically incompatible constructs with limited design flexibility. Alternatively, strategies involving the direct printing of bioinks onto polymer grids frequently suffer from delamination due to poor interfacial bonding between materials.^[^ ^9a^ ^]^ This study addresses these limitations by enabling stable bioink‐polymer integration. Notably, the distinct mechanical properties of each pattern were preserved after embedding within collagen‐based bioinks, confirming the feasibility of structural reinforcement through geometric integration.

The hybrid biopatches demonstrated marked improvements in mechanical performance, including increased elastic modulus, ultimate tensile strength, and fatigue resistance, compared to those of the collagen‐only biopatches. Furthermore, both chiral and chevron hybrid constructs maintained over 95% elastic energy retention and exhibited minimal strain loss after 50 loading‐unloading cycles, indicating that geometric reinforcement substantially enhances mechanical durability. This robustness positions the hybrid biopatches as promising candidates for clinical applications that demand reliable mechanical performance under dynamic physiological loading.

Preliminary in vivo results further underscore the benefits of mechanical reinforcement. The collagen‐only biopatch failed to remain attached to the defect site post‐implantation, whereas hybrid biopatches containing polymeric patterns remained stably fixed. This suggests that integrating polymeric frameworks not only strengthens the mechanical properties of the construct but also improves physical anchorage to host tissues via suture fixation. Enhanced mechanical coherence likely prevented early detachment, enabled stable retention at the wound site, and supported effective suture integration. This stable interface may also facilitate the directed migration and localized delivery of stem cells to the defect, thereby amplifying the regenerative potential of the implanted construct.^[^ ^10^, ^16^ ^]^

The regenerative outcomes observed in the porcine mucosal defect model further emphasize the significance of mechanical compatibility in tissue healing. Histological and immunofluorescence analyses indicated that chiral‐patterned biopatches promoted superior epithelial repair than both chevron‐patterned and untreated controls. These findings support the notion that mechanical compatibility between the implanted construct and host tissue contributes to improved healing responses.^[^ ^17^ ^]^ Given that both oral mucosa and tongue tissues are composed of irregular connective tissue, the enhanced regenerative outcomes in the chiral group align with the hypothesis that pattern‐specific mechanical matching promotes tissue repair. However, this association remains correlative, since this study did not include direct evaluation in regular connective tissues, such as tendons or ligaments, highlighting the need for further investigation.

To build upon these findings, future studies should explore the application of this hybrid biopatch platform across a broader range of connective tissues, particularly those with highly aligned architectures. This includes tissues such as tendons and ligaments, where anisotropic mechanical demands are critical.^[^ ^12a^ ^]^ Specifically, the dimensions of each geometric pattern, including unit cell size, strut thickness, and angular orientation, were selected based on preliminary printing feasibility and mechanical screening, ensuring structural stability and consistent material input across all designs.^[^ ^14^, ^18^ ^]^ The primary focus of this study was to investigate how distinct geometric patterns, when applied under standardized fabrication and material conditions, influence the delivery and therapeutic potential of stem cells. However, we acknowledge that a more comprehensive parametric optimization through numerical modeling is necessary to fully understand the structure–function relationship. In future studies, we plan to perform design optimization for selected patterns by systematically varying parameters such as line width, angular orientation, and strut thickness. Evaluating long‐term outcomes, such as tissue remodeling, integration, and functional restoration, would provide a more comprehensive understanding of the translational potential of this approach. Especially, it should be noted that the PCL framework used in this study may undergo gradual degradation over time, potentially leading to a reduction in mechanical integrity.^[^ ^19^ ^]^ Although this was not expected to affect the short‐term regenerative outcomes assessed in this study, future designs may benefit from incorporating composite or layered architectures, or utilizing slower‐degrading materials, to ensure sustained delivery of mechanical cues during extended tissue remodeling periods. Although stemness marker expression declined by Day 5 in vitro, the observed in vivo regenerative efficacy suggests that short‐term stem cell activity, particularly via paracrine mechanisms, may be sufficient to initiate tissue repair. Future studies involving lineage tracing or long‐term engraftment analysis will be needed to clarify the role of persistent stemness in sustained tissue regeneration. In addition, elucidating the biological mechanisms involved, such as cellular mechanotransduction and ECM remodeling within geometrically patterned microenvironments, would help clarify how mechanical cues influence healing at the molecular level.^[^ ^17^, ^20^ ^]^ Incorporating other adult or tissue‐specific stem cell types may also broaden the applicability of the platform across diverse regenerative contexts. Furthermore, the integration of spatially controlled biochemical signals, such as localized delivery of growth factors or matrix‐degrading enzymes, could further enhance the regenerative capacity of future iterations of the hybrid biopatch.

Collectively, these findings highlight the potential of geometry‐guided mechanical tuning as a robust design strategy for next‐generation bioengineered implants. By replicating the mechanical behavior of native tissues, hybrid biopatches may offer not only mechanical compatibility but also biologically supportive environments that facilitate tissue regeneration, particularly in applications involving dynamic mechanical loading.

## 4 Conclusion

This study introduced a hybrid biopatch platform that integrates stem cell‐laden collagen bioinks with mechanically tunable polymeric patterns fabricated via 3D printing. By varying the geometric design of these patterns, the platform successfully recapitulated the anisotropic and isotropic mechanical behavior characteristic of regular and irregular connective tissues, respectively. Specifically, the chiral pattern exhibited quasi‐isotropic compliance suitable for irregular tissues, whereas the chevron pattern demonstrated pronounced directional stiffness, aligning with the mechanical profile of regular connective tissues.

Mechanical testing confirmed that these properties were preserved after embedding the patterns within soft hydrogel matrices, resulting in significantly enhanced stiffness, extensibility, and fatigue resistance compared with those of unpatterned constructs. In vivo assessments further demonstrated that chiral hybrid biopatches promoted superior epithelial regeneration, supporting the hypothesis that mechanical compatibility between implant and host tissue can influence regenerative outcomes.

Collectively, these findings underscore the promise of geometry‐guided mechanical design as a versatile strategy for engineering tissue‐specific regenerative biomaterials. The proposed hybrid biopatch system offers a modular and tunable framework for developing bioengineered implants tailored to the mechanical demands of their target environments, advancing the potential of soft tissue repair and functional tissue engineering.

## 5 Experimental Section

### 3D Printing of Geometric Patterns and Collagen‐based Bioink

A 3D bioprinter (U‐FAB ACTIVO, CLECELL Co., Ltd., South Korea) was used to fabricate four geometric patterns commonly applied under static and dynamic loading conditions. A thermosensitive synthetic polymer, polycaprolactone (PCL; Mw = 43000–50000; Polysciences Inc., USA), was loaded into a 10 mL stainless steel syringe (Musashi Engineering Inc., Japan) and heated to 100 °C. The molten PCL was extruded at a constant speed of 300 mm min^−1^ along predefined printing paths through a nozzle with an inner diameter of 200 µm, under a pneumatic pressure of 500 kPa. A 1.5% collagen‐based bioink was prepared using decellularized porcine dermis to ensure biocompatibility and physiological relevance.^[^ ^21^ ^]^ The bioink was dispensed through a 26‐gauge needle (Musashi Engineering Inc., Japan) under a pneumatic pressure of approximately 15 kPa, using a printing speed of 250 mm min^−1^. This setup ensured stable extrusion and high‐fidelity patterning suitable for fabricating soft tissue constructs.

### Mechanical Analysis

To evaluate mechanical properties, five geometric patterns (lattice, honeycomb, chiral, chevron, and transverse chevron) were printed, with all patterns standardized to the same weight. Polymeric specimens were fabricated with a width of 20 mm, a length of 40 mm, and a thickness of 0.2 mm (single layer). For hybrid biopatches incorporating polymeric patterns, specimens with the same width and length but a thickness of 1 mm were used.

All samples were tested using a universal testing machine (UTM; LLOYD LS1, Ametek Inc., USA) equipped with a 500 N load cell. The gauge length was set to approximately 25 mm, and the crosshead speed was fixed at 5 mm min^−1^. Tensile tests were conducted until specimen failure. From the resulting stress–strain curves, the elastic modulus (E) was calculated as the slope within the initial 2% strain region. For cyclic testing, loading‐unloading conditions were based on the tensile results. Repeated loading‐unloading cycles were applied, up to 50 cycles or until specimen failure, to evaluate the durability and elastic recovery of the samples.

### Rheological Analysis

The rheological properties of collagen‐based bioinks at varying concentrations were analyzed using an advanced rheometric expansion system (Discovery HR‐20, TA Instruments Inc., USA) with a 20 mm parallel plate geometry. The system was maintained at 15 °C prior to testing to prevent premature gelation of the bioinks. A volume of 250 µL of each bioink was loaded onto the plate, with the gap set to 1 mm.

A temperature sweep test was conducted to evaluate gelation kinetics by measuring the complex modulus (G*) as the temperature increased from 15 °C to 37 °C at a rate of 5 °C min^−1^. Following gelation, which was induced by incubating the bioinks at 37 °C for 30 min, a dynamic frequency sweep test was performed to assess frequency‐dependent viscoelastic properties. Storage and loss moduli were measured over an angular frequency range of 0.1–100 rad s^−1^ at a constant strain of 1%.

### TMSC Isolation and Cell Culture

The TMSC isolation protocol was approved by the Institute of Laboratory Animal Resources (Approval No. PNU‐2008‐0001) at Pusan National University Hospital. Informed consent was obtained from all patients. TMSCs were isolated as previously described.^[^ ^13a^ ^]^ Discarded tonsillar tissues were obtained from pediatric patients who underwent tonsillectomy at the Department of Otorhinolaryngology, Pusan National University Hospital (Pusan, Republic of Korea). The tissues were mechanically minced and enzymatically digested using collagenase type I and DNase at 37 °C for 30 min. The digested mixture was filtered to isolate adherent mononuclear cells, which were cultured in Dulbecco's Modified Eagle's Medium (DMEM; Gibco, Grand Island, NY, USA) supplemented with 10% fetal bovine serum (Gibco) and 1% penicillin/streptomycin. After 48 h, non‐adherent cells were removed, and the adherent TMSCs were maintained in a humidified incubator at 37 °C with 5% CO_2_. Once cultures reached a confluency of approximately 85%, cells were detached using trypsin‐ethylenediaminetetraacetic acid (trypsin‐EDTA; Merck, USA) and either subcultured or used in experiments. All TMSCs used in this study were within passage 5.

### Cell Viability and Proliferation Analysis

TMSC viability was assessed using a Live/Dead Viability/Cytotoxicity Kit (Invitrogen, Carlsbad, CA, USA), following the manufacturer's instructions. Briefly, samples were washed with phosphate‐buffered saline (PBS) and incubated in a staining solution containing calcein acetoxymethyl (calcein‐AM) and ethidium homodimer‐1 at 37 °C for 20 min. Fluorescent images were acquired using a fluorescence microscope (Axio Zoom, Zeiss, Germany).

TMSC proliferation was evaluated using a Cell Counting Kit‐8 (CCK‐8; Sigma‐Aldrich, USA), following the manufacturer's protocol. Samples were gently rinsed with PBS and treated with CCK‐8 reagent diluted in DMEM for 3 h in a humidified incubator at 37 °C. The absorbance, corresponding to cell metabolic activity, was measured at 450 nm using a microplate reader (Asys UVM 340; Biochrom, UK). All measurements were performed in triplicate.

### Gene Expression Analysis

Quantitative reverse transcription‐polymerase chain reaction (qRT‐PCR) was performed to assess gene expression levels among the experimental groups. Total RNA was extracted using TRIzol reagent (Invitrogen), in accordance with the manufacturer's instructions. RNA concentration and purity were determined using a NanoDrop spectrophotometer (ND‐2000; Thermo Fisher Scientific, USA). Complementary DNA (cDNA) was synthesized using a commercial cDNA synthesis kit (Thermo Fisher Scientific).

Quantitative PCR was performed using SYBR Green Master Mix (Thermo Fisher Scientific) and a 7500 Real‐Time PCR System (Applied Biosystems, USA). Primers were designed based on sequences from the National Center for Biotechnology Information database (Table S1, Supporting Information). Gene expression levels were normalized to those of glyceraldehyde‐3‐phosphate dehydrogenase (*GAPDH*), and relative expression was calculated using the 2^−ΔΔCt^ method.

### In Vivo Evaluation of Hybrid Biopatches

In vivo studies were conducted using three pigs (30–40 kg) from Yorkshire, Landrace, and Duroc breeds, with approval from the Institutional Animal Care and Use Committee of Pusan National University Yangsan Hospital (Approval No. P2024‐009‐A1C0). After an overnight fast, general anesthesia was induced via intramuscular injection of ketamine hydrochloride (20 mg kg⁻¹) and xylazine (2 mg kg⁻¹), and maintained via inhalation of 3% isoflurane. Animals were intubated using a reinforced EMG endotracheal tube (7.0 mm; Medtronic Xomed Inc.), and correct placement was confirmed by laryngoscopy. Upon visual identification of the recurrent laryngeal and vagus nerves, electrical stimulation was applied using a handheld probe, and evoked electromyographic signals were recorded (NIM‐Response 3.0 system, Medtronic Xomed Inc.). Following anesthetic preparation, full‐thickness mucosal defects (10 mm × 10 mm) were surgically created at two anatomical sites: the oral mucosa (inner cheek) and the lateral tongue. Three defects were created per animal at each site. Chiral and chevron hybrid biopatches were implanted into one defect per site, while the third was left untreated as a control. The biopatches were gently positioned and fixed at four quadrants using interrupted 5‐0 Vicryl sutures (Ethicon, USA), ensuring stable placement without wrinkling or excessive tension. All procedures were performed by a board‐certified oral and maxillofacial surgeon to ensure anatomical accuracy and clinical relevance. Surgical sites were irrigated with sterile saline, and animals were allowed to recover under close monitoring. Postoperative care included systemic antibiotics (cefazolin, 25 mg kg⁻¹ for 3 days) and soft diet feeding to minimize wound disruption.

### Histological Analysis

Experimental samples were fixed in 4% paraformaldehyde (Biosesang, Republic of Korea) for 2 h and washed three times with PBS. Fixed tissues were dissected for histological processing.

For histological staining, samples were embedded in paraffin wax and sectioned to a thickness of 30 µm using a microtome (Leica Biosystems Company, Germany). Sections were treated with 1% bovine serum albumin (Merck) for 1 h to block nonspecific binding sites. After deparaffinization, H&E staining was performed using a commercial kit (Abcam), following the manufacturer's instructions. Stained slides were scanned using an automated digital slide scanner (Pannoramic MIDI; 3DHISTECH, Hungary).

For immunostaining, fixed samples were embedded in optimal cutting temperature compound within a cryomold and flash‐frozen in liquid nitrogen. Cryosections were incubated overnight at 4 °C with primary antibodies against cytokeratin 10 (K10; 1:100; Invitrogen) and cytokeratin 15 (K15; 1:100; Invitrogen). Alexa Fluor‐conjugated secondary antibodies (1:1000; Invitrogen) and DAPI (1:1000; Vector Laboratories, Burlingame, CA, USA) were applied for 30 min at room temperature. Immunostained sections were imaged using a confocal microscope (LSM 880; Carl Zeiss, Germany).

### Statistical Analysis

Quantitative data were presented as mean ± standard deviation (SD). A two‐tailed Student's t‐test was used for comparisons between two groups. For comparisons among three or more groups, one‐way analysis of variance followed by Tukey's post hoc test was performed. Differences were considered statistically significant at *p* < 0.05. All experiments were conducted in quadruplicate (n = 4).

## Conflict of Interest

The authors declare no conflict of interest.

## Supporting information



Supporting Information

## Data Availability

The data that support the findings of this study are available from the corresponding author upon reasonable request.
