# Supplementary material for: 3D Bioprinting‐Assisted Engineering of Stem Cell‐Laden Hybrid Biopatches With Distinct Geometric Patterns Considering the Mechanical Characteristics of Regular and Irregular Connective Tissues
Source: Adv Healthc Mater. 2025 Jul 7;14(25):2502763. doi: 10.1002/adhm.202502763 (PMC12477570; doi:10.1002/adhm.202502763)
Supplement: Supplementary file 1 — Supporting Information [file ADHM-14-0-s001.docx]

Supporting Information

3D Bioprinting-Assisted Engineering of Stem Cell-Laden Hybrid Biopatches with Distinct Geometric Patterns Considering the Mechanical Characteristics of Regular and Irregular Connective Tissues

Minjun Ahn, Gyu-Tae Park, Arvind Kumar Shukla, Boguen Kwon, Jae-Ho Kim, Eui-Suk Sung*, and Byoung Soo Kim*


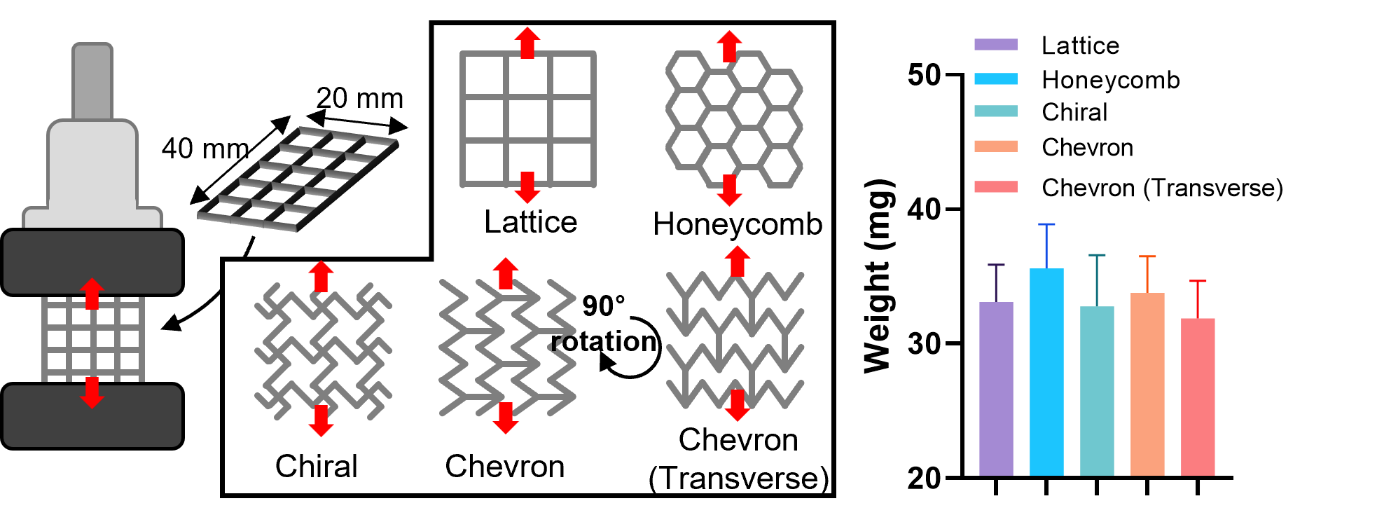


Figure S1. Standardized preparation of 3D printed polymeric patterns for mechanical testing.


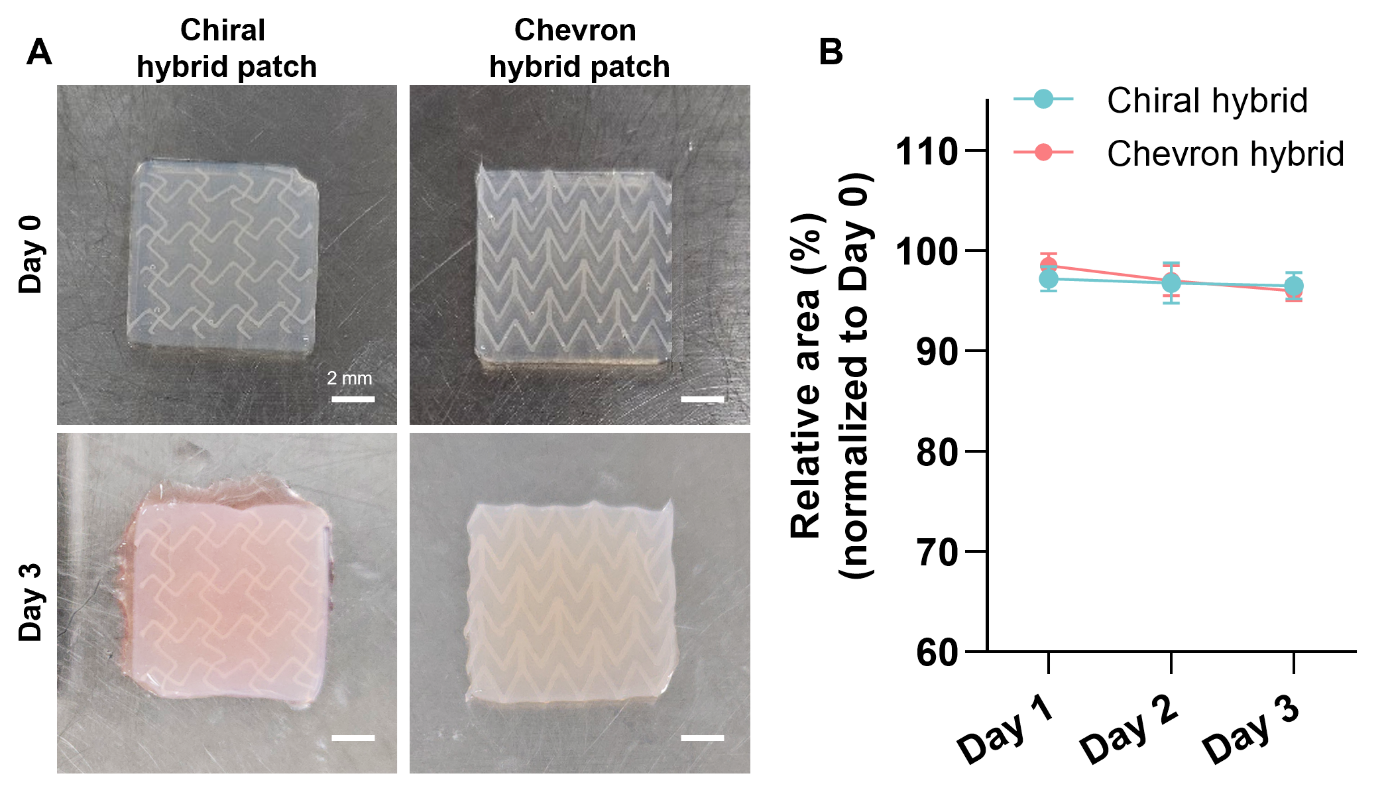


Figure S2. Contraction behavior of chiral- and chevron-incorporating hybrid biopatches. (A) Representative photographs of chiral and chevron hybrid biopatches at day 0 and day 3. Scale bars: 2 mm. (B) Quantification of construct area over time, normalized to that on day 0, showing minimal contraction in both designs.

**Table S1.** Sequences of primers used for qRT-PCR analysis.

| **Gene** | **Sequence** | |
| --- | --- | --- |
| **Glyceraldehyde-3-phosphate dehydrogenase (GAPDH)** | Forward | GTCTCCTCTGACTTCAACAGCG |
|  | Reverse | ACCACCCTGTTGCTGTAGCCAA |
| **Octamer-binding protein 4**  **(OCT4)** | Forward | CCTGAAGCAGAAGAGGATCACC |
|  | Reverse | AAAGCGGCAGATGGTCGTTTGG |
| **SRY-Box Transcription Factor 2 (SOX2)** | Forward | GCTACAGCATGATGCAGGACCA |
|  | Reverse | TCTGCGAGCTGGTCATGGAGTT |
| **Nanog homeobox**  **(NANOG)** | Forward | GTCTGCGGAGGTGGTTCCTCT |
|  | Reverse | CTCCAACATCCTGAACCTCAGC |
